# Differentially abundant proteins, metabolites, and lipid molecules in spaghetti meat compared to normal chicken breast meat: Multiomics analysis

**DOI:** 10.1016/j.psj.2025.105165

**Published:** 2025-04-14

**Authors:** Janghan Choi, Majid Shakeri, Brian Bowker, Hong Zhuang, Byungwhi Kong

**Affiliations:** US National Poultry Research Center, USDA-ARS, Athens, GA 30605, USA

**Keywords:** Chicken breast myopathies, Spaghetti meat, Multiomics, Nicotinamide adenine dinucleotide, Carnitines, Triglycerides

## Abstract

Spaghetti meat (SM), a recently emerging muscle myopathy in chicken breast meat, is characterized by a loss of muscle fiber integrity, resulting in a spaghetti-like appearance**.** Understanding the differences in proteins, metabolites, and lipids through a multiomics approach in SM can identify its quality traits and elucidate its exact causes. The purpose of this study was to investigate differentially abundant proteins, metabolites, and lipid molecules in SM compared to normal chicken breast meat (Control). The supernatant from sample homogenates was subjected to ultra-high performance liquid chromatography (UHPLC) analysis for multiomic profiling. A total of 16 chicken breast fillets (*Pectoralis major*) representing Control (*n* = 8) and SM (*n* = 8) groups were collected from a commercial slaughterhouse. A total of 2593 molecules were identified and composed of 1903 proteins, 506 lipids, 181 compounds and 3 electrolytes. There were 632 differential molecules composed of 503 proteins, 76 lipids, 50 metabolites, and 3 electrolytes. In comparing SM and Control, the protein, metabolite, and lipid molecules with the greatest fold change were calponin, decanoylcarnitine, and ceramide [N‑hydroxy-sphingosine] (Cer[NS]) d18:1_26:1, respectively. Plasmenylphosphatidylcholine (Plasmenyl-PC) and triglycerides (TG) were significantly decreased and increased, respectively, in SM compared to Control. Acylcarnitines (AC) were significantly decreased in SM compared to Control. Decanoylcarnitine, lauroylcarnitine, linoleyl-carnitine, oleoyl-carnitine, hexanoylcarnitine were downregulated in SM compared to Control, and adenosine 5′-diphosphoribose and nicotinamide adenine dinucleotide (NAD) were downregulated in SM. Carbon metabolism, glycolysis/glucogenesis, ribosome, biosynthesis of amino acids, and aminoacyl-tRNA biosynthesis were selected in the top 10 enriched Kyoto Encyclopedia of Genes and Genomes (KEGG) pathways, identified by using differential proteins. Hence, SM had different proteins, metabolites, and lipid molecules related to β-oxidation, carbon and energy metabolism, lipid formation, and protein and amino acid metabolism compared to Control. Results from this study showed physiological alterations found in SM myopathy. Therefore, to mitigate SM in broilers, interventions should: 1) increase NAD and carnitines, 2) reduce triglycerides, and 3) modulate β-oxidation and energy metabolism via nutritional, genetic, or systemic approaches.

## Introduction

The productivity and efficiency of the broiler industry have been significantly enhanced through advanced genetic selection and optimized nutrition programs (Choi et al., 2023). These breakthroughs have made broiler meat a nutritious and affordable protein source worldwide. Broiler meat was the most consumed meat in the US in 2022 with approximately 45 kg per capita (Shahbandeh, 2023). However, breast myopathies including white stripping (WS), wooden breast (WB), and spaghetti meat (SM) are frequently observed in chicken breast meat in modern broiler production (Che et al., 2022). These breast myopathies negatively impact texture, water-holding capacity, and consumer acceptance, leading to tougher meat, reduced juiciness, poor appearance, and decreased marketability of affected products (Bowker and Zhuang, 2016; Choi et al., 2024b; Tasoniero et al., 2020).

To decrease the incidence and severity of breast myopathies in broiler chickens, it has been crucial to gain a deeper understanding of the underlying quality defects and fully unravel the etiology of these conditions. Due to the lack of precise knowledge regarding the factors that induce breast myopathies, a variety of omics techniques have been employed, offering significant advantages in identifying previously unknown causes and mechanisms inducing these disorders (Zhang et al., 2023). Despite the extensive use of transcriptomics (Che et al., 2024; Choi et al., 2025), proteomics (Bottje et al., 2021; Kong et al., 2024), metabolomics (Boerboom et al., 2022; Choi et al., 2024a; Wang et al., 2023), and lipidomics (Liu et al., 2022) to elucidate the etiology and quality traits of WS or WB, the exact causes of these muscle myopathies remain unclear. Multiomics, integrating proteomics, metabolomics, and lipidomics, offers valuable insights into overall biochemical changes associated with complex traits (Subramanian et al., 2020). Multiomics could potentially offer valuable insights into breast myopathies research (Zhou et al., 2025).

SM, a recently identified muscular abnormality in chicken breast meat, is characterized by a loss of muscle fiber integrity, resulting in a spaghetti-like appearance (Baldi et al., 2021). The incidence rate of SM could reach up to 20 %, but not many studies have been conducted on SM due to its recentness (Baldi et al., 2021). SM can negatively affect consumer acceptance by causing visual defects and impacting key quality attributes, such as water holding capacity, color, and nutrient values (Tasoniero et al., 2020). Similar to other breast myopathies, the etiologies and quality characteristics of SM remain poorly understood. Understanding the differences in proteins, metabolites, and lipids through a multiomics approach in SM can be for elucidating its exact causes and identifying its quality traits. To the best of our knowledge, no study has used a multiomics approach to characterize SM. Therefore, the purpose of this study was to investigate differentially abundant protein, metabolite, and lipid molecules through a multiomics approach in SM compared to normal chicken breast meat (Control).

## Materials and methods

### Experimental design, multiomic analysis, and data processing

**Fig. 1:**
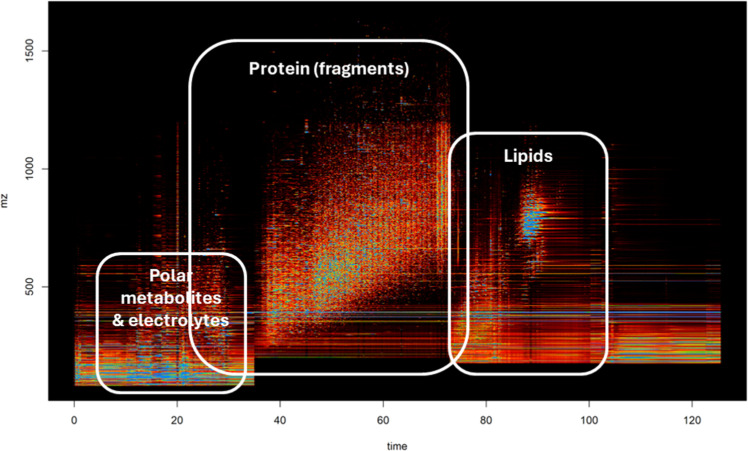
Illustration of detection of different molecules including protein, metabolites, lipids, and electrolytes in chicken breast meat using LC/MS.

A total of 16 chicken breast fillets (*Pectoralis major*) representing Control (*n* = 8) and SM (*n* = 8) groups were collected at 8 wks of age from a commercial slaughterhouse in Georgia, USA. Chicken breast fillets without any myopathies (WS, WB, or SM), and chicken breast fillets with severe symptoms of SM were selected for the Control and SM groups, respectively. The middle portion of the cranial end of the breast fillets were cut into 1 cm × 1 cm × 1 cm samples and stored at −80°C until further analysis. Samples were shipped with dry ice to a commercial research laboratory (Dalton Bioanalytics Inc., Los Angeles, CA) for protein, lipid, and small molecule multiomic analysis. The multiomic analysis using liquid chromatography mass spectrometry (LC/MS) was conducted based on methods described in a previous study (Altendahl et al., 2022). Briefly, 30 mg of muscle samples were homogenized with internal standards, buffer, and ethanolic water using a bead mill. A quality control (QC) sample was prepared from a mixture of all sample extracts to examine the reproducibility of the analysis. Afterwards, trypsin was added and homogenates were digested for 2 h at 37°C. To stop the digestion precipitation was induced by adding ethanol and the samples were centrifuged at 14,000 × *g* for 5 min. The collected supernatant was analyzed on an ultra-high performance liquid chromatography (UHPLC) instrument with microflow mixed mode. The mobile phase additives were formic and ammonium acetate. Hydrophilic, peptide, and lipophilic compounds were observed at 0 to 35 min, 35 to 73 min, and 73 to 100 min, respectively (Fig. 1). Identification was performed using Full scan with data dependent MS2 (ddMS2) acquisition with an identification library (peptide/protein ID: MSFragger software; Lipid ID: LipiDex software; Metabolite ID: Compound Discoverer and Dalton's internal Metabodex). Metabolites were identified using Compound Discoverer library and Dalton's internal Metabodex. Quantification was performed using MS1 BoxCar acquisition (msx-tSIM). Principal component analysis (PCA) was conducted using the base package of R software (version 4.1.2) with parameter scale=True indicating unit variance (UV) scaling for normalizing the data. A dendrogram was generated according to the relative intensity of the differential metabolites using R software. A volcano plot was generated using -log_10_(*P* value) and log_2_(fold change) with GraphPad Prism (Version 9.1.0; GraphPad Software, San Diego, CA). The top 25 up- and downregulated molecules with the greatest fold change and *P* < 0.05 without uncharacterized molecules were selected. Differential proteins, metabolites, and lipids (*P* < 0.05) were subjected to gene ontology (GO), Kyoto Encyclopedia of Genes and Genomes (KEGG) enrichment, and REACTOME pathway enrichment analyses. Protein and metabolite enrichment assays were conducted using WebGestalt (https://www.webgestalt.org/) and MetaboAnalyst (https://new.metaboanalyst.ca/), respectively. Lipid enrichment was conducted using the in-house method by Dalton Bioanalytics Inc., and the top 10 pathways with the lowest *P* value were selected.

### Validation assays

Selected metabolites showing significantly greater differences were analyzed using commercial kits as follows: Lactate assay kit (Cat. No. MAK064, Sigma-Aldrich, St. Louis, MO); nicotinamide adenine dinucleotide (NAD)/ NAD + hydrogen (NADH) quantification kit (Cat. No. MAK037, Sigma-Aldrich); triglycerides (TG) colorimetric assay kit (Item No. 10010303, Cayman Chemical, Ann Arbor, MI). The concentrations of the metabolites of each sample were normalized with the protein concentration of each sample measured using a Thermo Scientific™ Pierce™ BCA Protein Assay Kits (Cat. no T1949-500ML, Sigma-Aldrich).

Selected proteins (FHL1: four and a half LIM domains and JPH2: junctophilin 2) were analyzed using a Simple Western system (ProteinSimple, Bio-Techne, Minneapolis, MN). The 4 ug of total protein lysates from SM and Control were incubated with rabbit anti-FHL1 antibody (Cat. no AV34378, Sigma-Aldrich) at a 1:1,000 dilution (∼44 kDa) or rabbit anti-JPH2 antibody (Cat. no AV51358, Sigma-Aldrich) at a 1:1,000 dilution (∼74 kDa) and rabbit antibody system control (Cat. No. 042-196, Bio-Techne) with protein normalization. Compass software (Bio-Techne) normalized peaks against the lowest peak area detected on a capillary.

### Statistical analysis

Statistical comparisons of differential molecules from the multiomic assay, as well as selected metabolites and proteins from the validation assays, were performed between the Control and SM groups using an unpaired t-test in GraphPad Prism (Version 9.1.0; GraphPad Software, San Diego, CA).

## Results

### Number of the differential molecules and multivariate analyses

A total of 2593 molecules were identified and composed of 1903 proteins, 506 lipids, 181 compounds and 3 electrolytes (Table 1). There were 632 differential molecules (up: 265 and down: 367) composed of 503 proteins (up: 225 and down: 278), 76 lipids (up: 25 and down: 51), 50 metabolites (up: 14 and down: 36), and 3 electrolytes (up: 1 and down: 2). There were 336 differential molecules (up: 167 and down: 169) with a greater than 1.5-fold change composed of 245 proteins (up: 132 and down: 113), 58 lipids (up: 24 and down: 34), and 33 metabolites (up: 11 and down: 22).

**Table 1:** Number of identified and differential protein, lipid, metabolite, and electrolyte molecules (*P* < 0.05) with a greater than 1.5-fold change of spaghetti meat compared with normal chicken breast meat.

		Differential molecules (*P* < 0.05)			*P* < 0.05 with > 1.5-fold change		
	Identified	Total	Up	Down	Total	Up	Down
Total	2593	632	265	367	336	167	169
Proteins	1903	503	225	278	245	132	113
Lipids	506	76	25	51	58	24	34
Metabolites	181	50	14	36	33	11	22
Electrolytes	3	3	1	2	0	0	0

**Fig. 2:**
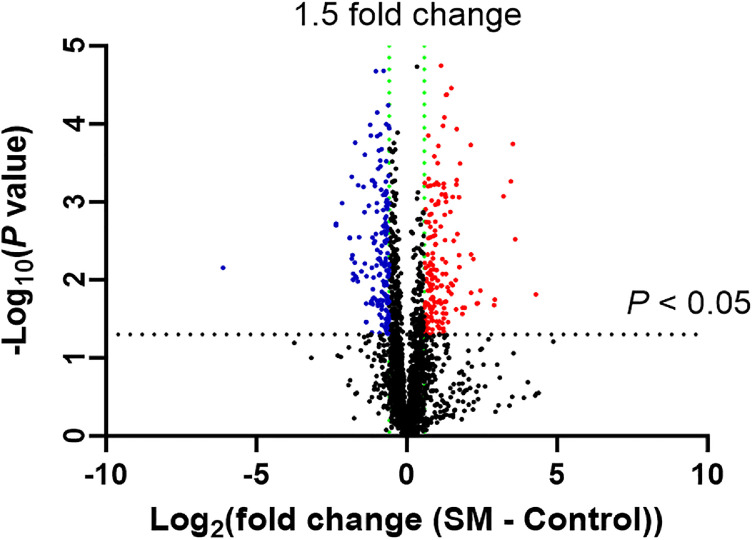
Volcano plot of different molecules including proteins, metabolites, lipids, and electrolytes for spaghetti meat (SM) compared with normal chicken breast meat (Control).

**Fig. 3:**
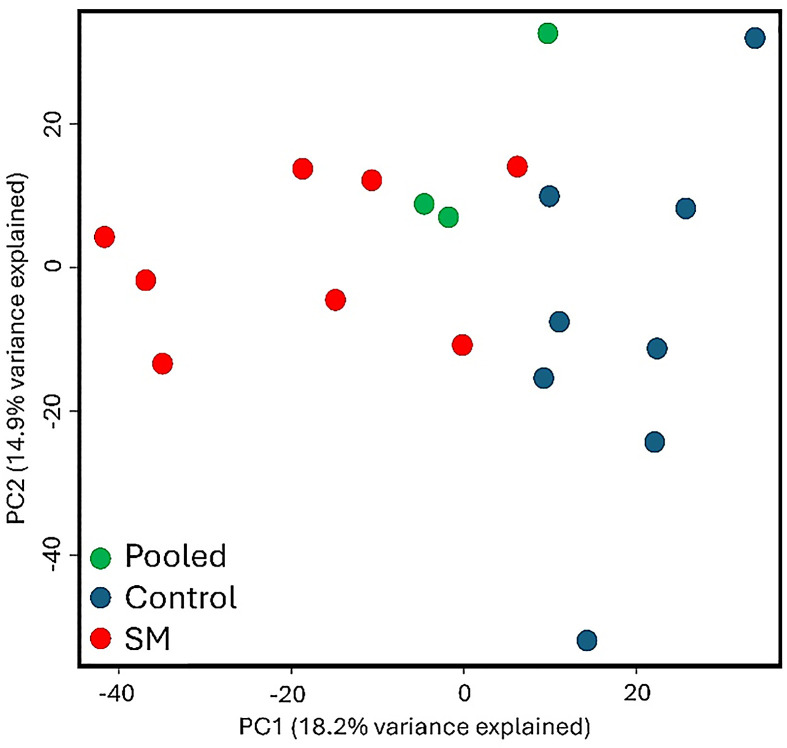
Principal component analysis of different molecules including proteins, metabolites, lipids, and electrolytes for spaghetti meat (SM) compared with normal chicken breast meat (Control). pool: pooled samples for quality control.

**Fig. 4:**
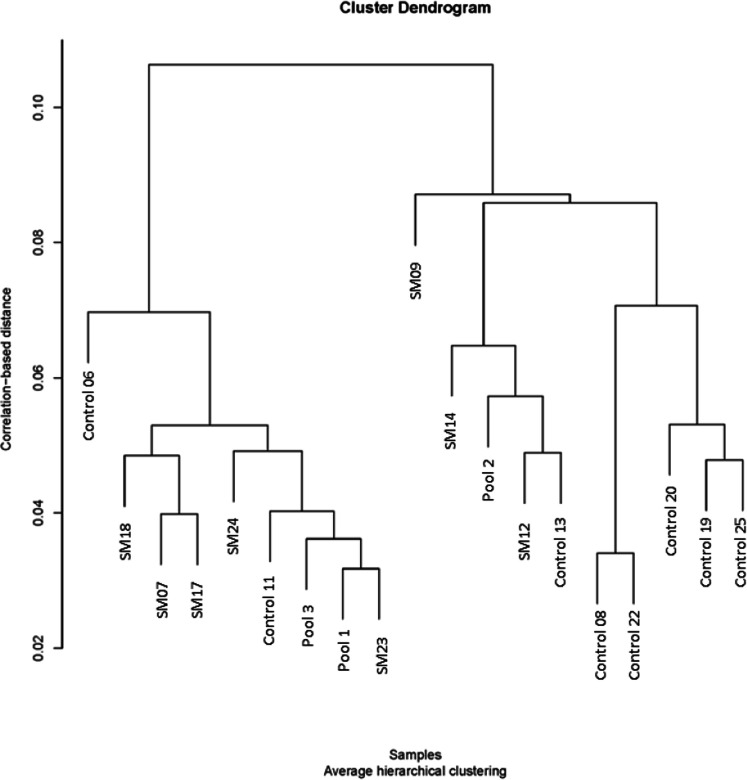
Cluster dendrogram of different molecules including proteins, metabolites, lipids, and electrolytes for spaghetti meat (SM) compared with normal chicken breast meat (Control). Pool: pooled samples for quality control.

As shown in Fig. 2, the volcano plot demonstrated differential molecules with a greater than 1.5-fold change. As shown in Fig. 3, the PCA plot showed that the Control and SM groups were separated, but the samples were dispersed. As shown in Fig. 4, the cluster dendrogram showed that the two groups were not clearly separated due to several samples (Control 06, 11 and SM 09, 14, 12). The pooled samples were in the middle of the groups in the PCA plot and cluster dendrogram, which suggests that the variations among the samples were attributed to biological differences.

### Differential molecules with high fold change

Table 2 shows the top 25 molecules with the greatest fold change in the comparison of SM and Control. While there were a greater number of identified and differential proteins, there were 10 proteins, 7 metabolites, and 8 lipids among the top 25 molecules with the greatest fold change. In comparing the SM and Control, the protein, metabolite, and lipid molecules with the greatest fold change were calponin, decanoylcarnitine, and ceramide [N‑hydroxy-sphingosine] (Cer[NS]) d18:1_26:1, respectively. Table 3, Table 4, Table 5, Table 6 showed the top 20 proteins, metabolites, lipids, and electrolytes, respectively, with the greatest fold change in the comparison of SM and Control. Adenosine triphosphate (ATP) synthase membrane subunit f and actin were significantly upregulated, but protein‑serine/threonine kinase was significantly lower in SM compared to Control. Decanoylcarnitine, lauroylcarnitine, linoleyl-carnitine, oleoyl-carnitine, hexanoylcarnitine were downregulated, and propionylcarnitine were increased in SM compared to Control. Acylcarnitines (AC) including AC10:0, AC12:0, AC20:2, AC18:2, AC16:1, and AC18:1 were downregulated in SM compared to Control. Adenosine 5′-diphosphoribose and nicotinamide adenine dinucleotide (NAD) were downregulated in SM compared to Control.

**Table 2:** Top 25 differential protein, metabolite, and lipid molecules with the greatest fold change regardless of up- or downregulation when spaghetti meat was compared with normal chicken breast meat.

	Molecules	Type of molecules	Log_2_(fold change)	*P* value
1	Calponin	protein	4.30	0.0154
2	SH3 domain-containing protein	protein	3.21	0.0008
3	Serine/threonine-protein phosphatase	protein	2.92	0.0178
4	Dimethylaniline monooxygenase [N-oxide-forming]	protein	2.91	0.0210
5	Cer[NS] d18:1_26:1	lipid	2.45	0.0137
6	Cer[NS] d18:1_26:2	lipid	2.36	0.0199
7	AC 10:0	lipid	−2.35	0.0019
8	Decanoylcarnitine	compound	−2.35	0.0020
9	Triethanolamine	compound	2.32	0.0201
10	Coatomer subunit epsilon	protein	2.22	0.0054
11	Octanoyl-l-Carnitine	compound	−2.15	0.0010
12	Muscle-restricted coiled-coil protein	protein	2.14	0.0146
13	ATP synthase membrane subunit f	protein	1.93	0.0228
14	AC 12:0	lipid	−1.90	0.0029
15	Lauroylcarnitine	compound	−1.90	0.0029
16	PC 18:4_26:0	lipid	1.86	0.0226
17	PG 18:1_20:4	lipid	1.85	0.0272
18	Acetylarginine	compound	−1.83	0.0005
19	Protein-serine/threonine kinase	protein	−1.80	0.0048
20	AC 20:2	lipid	−1.79	0.0083
21	Linoleyl-carnitine	compound	−1.78	0.0099
22	AC 18:2	lipid	−1.78	0.0099
23	Ankyrin repeat domain-containing protein 1	protein	1.77	0.0003
24	Propionylcarnitine	compound	1.75	0.0068
25	Aldolase_II domain-containing protein	protein	1.74	0.0009

SH3: Src homology 3; Cer[NS]: ceramide [N‑hydroxy-sphingosine]; AC: acylcarnitine; ATP: adenosine triphosphate; PC: phosphatidylcholine; PG: phosphatidylglycerol

**Table 3:** Top 20 differential proteins with the greatest fold change regardless of up- or downregulation when spaghetti meat was compared with normal chicken breast meat.

	Proteins	Protein ID	Log_2_(fold change)	*P* value
1	Calponin	Q5ZKU6	4.30	0.0154
2	SH3 domain-containing protein	A0A1L1RP17	3.21	0.0008
3	Serine/threonine-protein phosphatase	E1C2T7	2.92	0.0178
4	Dimethylaniline monooxygenase [N-oxide-forming]	A0A1D5PJW1	2.91	0.0210
5	Coatomer subunit epsilon	Q5ZIK9	2.22	0.0054
6	Muscle-restricted coiled-coil protein	R4GKM8	2.14	0.0146
7	ATP synthase membrane subunit f	A0A1D5P1J7	1.93	0.0228
8	Protein-serine/threonine kinase	A0A1D5PF79	−1.80	0.0048
9	Ankyrin repeat domain-containing protein 1	F1NYV8	1.77	0.0003
10	Small muscular protein	Q9UHP9	1.74	0.0048
11	Aldolase_II domain-containing protein	F1NEA1	1.74	0.0009
12	Endoplasmic reticulum junction formation protein lunapark	A0A1D5PNA3	−1.73	0.0060
13	Methyltransf_11 domain-containing protein	A0A1D5P5L5	−1.72	0.0002
14	N-acetyltransferase domain-containing protein	A0A1D5NYR0	1.69	0.0026
15	Tenascin	A0A1D5PJ88	1.66	0.0001
16	Actin, gamma-enteric smooth muscle	P63270	1.65	0.0006
17	4′-phosphopantetheine phosphatase	E1BV98	1.64	0.0238
18	Musculoskeletal embryonic nuclear protein 1	Q76MS9	1.62	0.0100
19	Transket_pyr domain-containing protein	A0A1D5NWL4	1.52	0.0009
20	VPS37 C-terminal domain-containing protein	F1NN84	1.52	0.0117

SH3: Src homology 3; ATP: adenosine triphosphate.

**Table 4:** Top 20 differential metabolites with the greatest fold change regardless of up- or downregulation when spaghetti meat was compared with normal chicken breast meat.

	Metabolites	Log_2_(fold change)	*P* value
1	Decanoylcarnitine	−2.35	0.0020
2	Triethanolamine	2.32	0.0201
3	Octanoyl-carnitine	−2.15	0.0010
4	Lauroylcarnitine	−1.90	0.0029
5	Acetylarginine	−1.83	0.0005
6	Linoleyl-carnitine	−1.78	0.0099
7	Propionylcarnitine	1.75	0.0068
8	Methylimidazoleacetic acid	−1.61	0.0006
9	Adenosine 5′-diphosphoribose	−1.58	0.0028
10	Trigonelline	−1.51	0.0078
11	Alanyltyrosine	−1.44	0.0006
12	Oleoyl-carnitine	−1.35	0.0346
13	Nicotinamide adenine dinucleotide	−1.22	0.0001
14	Arachidonoyl-carnitine	−1.11	0.0171
15	Hexanoylcarnitine	−1.00	0.0013
16	Spermidine	1.00	0.0126
17	N-omega-acetylhistamine	0.96	0.0035
18	N-acetyl-histamine	0.96	0.0035
19	Arecoline	−0.91	0.0087
20	Taurine	0.91	0.0073

**Table 5:** Top 20 differential lipids with the greatest fold change regardless of up- or downregulation when spaghetti meat was compared with normal chicken breast meat.

	Lipids	Log_2_(fold change)	*P* value
1	Cer[NS] d44:2	2.45	0.0137
2	Cer[NS] d44:3	2.36	0.0199
3	AC 10:0	−2.35	0.0019
4	AC 12:0	−1.90	0.0029
5	PC 44:4	1.86	0.0226
6	PG 38:5	1.85	0.0272
7	AC 20:2	−1.79	0.0083
8	AC 18:2	−1.78	0.0099
9	AC 14:1	−1.70	0.0089
10	FA 14:1	−1.67	0.0094
11	AC 16:1	−1.63	0.0104
12	LysoPC 20:0	1.47	0.0111
13	PC 41:4	−1.40	0.0062
14	Cer[NDS] d44:2	1.36	0.0124
15	FA 14:0	−1.35	0.0200
16	AC 18:1	−1.35	0.0345
17	PC 33:0	1.31	0.0188
18	PG 38:4	1.29	0.0059
19	HexCer[NS] d34:1	1.26	0.0015
20	PC 42:3	1.22	0.0418

AC: Acylcarnitine; PC: phosphatidylcholine; PG: Phosphatidylglycerol; FA: fatty acid; LysoPC: Lysophosphatidylcholines; Cer[NDS]: Ceramide [N-acyl-sphingosine]; HexCer[NS]: Hexosylceramide [N‑hydroxy-sphingosine].

**Table 6:** Differential electrolytes when spaghetti meat was compared with normal chicken breast meat.

	Electrolytes	Log_2_(fold change)	*P* value
1	Potassium	−0.42	0.0008
2	Magnesium^2+^	−0.37	0.0203
3	Zinc^2+^	0.48	0.0423

### Enrichment analyses

**Fig. 5:**
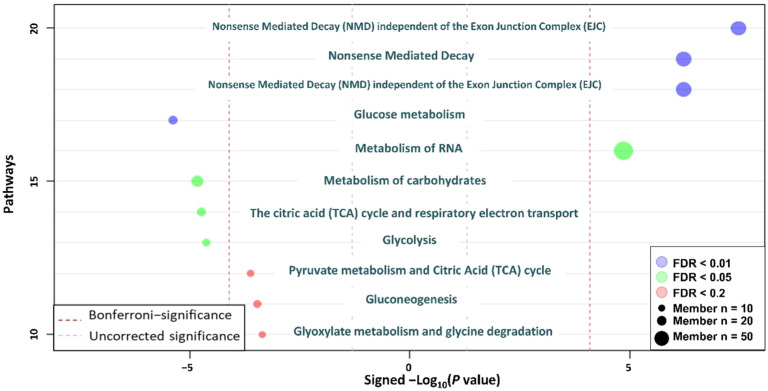
Reactome pathway enrichment analysis of different molecules including proteins, metabolites, lipids, and electrolytes for spaghetti meat (SM) compared with normal chicken breast meat (Control).

**Fig. 6:**
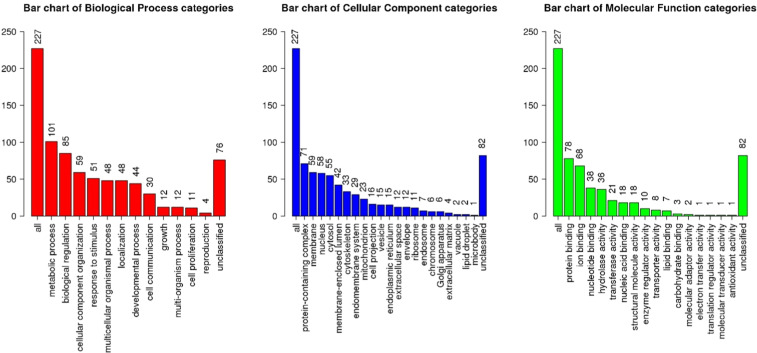
Gene ontology using differential molecules including proteins, metabolites, lipids, and electrolytes for spaghetti meat (SM) compared with normal chicken breast meat (Control).

Table 7 lists the top 10 KEGG pathways with the most significant *P* values identified using differential proteins. The carbon metabolism pathway had the lowest *P* value among the enriched KEGG pathways identified using differential proteins. Carbon metabolism, glycolysis/glucogenesis, ribosome, biosynthesis of amino acids, and aminoacyl-tRNA biosynthesis were selected in the top 10 enriched KEGG pathways identified using differential proteins. Table 8 lists KEGG pathways with significant *P* values (*P* < 0.05) identified using differential metabolites. The histidine metabolism pathway had the lowest *P* value among the enriched KEGG pathways identified using differential metabolites and pyruvate metabolism and glycolysis/glucogenesis were different between SM and Control. Table 9 listed results of lipid classification with significant differences (*P* < 0.05) identified using differential lipids. Plasmenylphosphatidylcholine (Plasmenyl-PC), TG, and AC were the most significantly different lipid classes annotated. Plasmenyl-PC and TG were significantly decreased and increased, respectively, in SM compared to Control. AC was significantly decreased in SM compared to Control. Fig. 5 shows the pathway enrichment analysis (Reactome) of differential molecules, including proteins, metabolites, and lipids in spaghetti meat (SM) compared to Control. Nonsense-mediated decay was the most enriched pathway, while pathways related to carbohydrate metabolism were also highly represented. Gene ontology (GO) assay demonstrated that diverse pathways associated with biological process, cellular component, and molecular function were influenced in SM compared to Control (Fig. 6).

**Table 7:** Top 10 Kyoto Encyclopedia of Genes and Genomes (KEGG) enriched pathways with the lowest *P* values (*P* < 0.05) performed using differential proteins (*P* < 0.05).

Gene Set	Description	Size	Expect	Ratio	*P* value
gga01200	Carbon metabolism	94	1.86	8.05	<0.0001
gga00010	Glycolysis / Gluconeogenesis	49	0.97	11.32	<0.0001
gga03010	Ribosome	107	2.12	4.71	<0.0001
gga00030	Pentose phosphate pathway	26	0.52	9.70	0.0001
gga00500	Starch and sucrose metabolism	28	0.56	9.01	0.0002
gga00051	Fructose and mannose metabolism	32	0.63	7.88	0.0004
gga00620	Pyruvate metabolism	35	0.69	7.21	0.0006
gga01230	Biosynthesis of amino acids	56	1.11	5.40	0.0008
gga00020	Citrate cycle (TCA cycle)	24	0.48	8.41	0.0011
gga00970	Aminoacyl-tRNA biosynthesis	35	0.69	5.77	0.0047

**Table 8:** Kyoto Encyclopedia of Genes and Genomes (KEGG) enriched pathways with significant *P* values (*P* < 0.05) performed using differential metabolites (*P* < 0.05).

Pathways	Significant metabolites/total related metabolites	*P* value
Histidine metabolism	4/16	0.0000
beta-Alanine metabolism	4/21	0.0001
Arginine and proline metabolism	3/36	0.0072
Pyruvate metabolism	2/23	0.0275
Glycolysis / Gluconeogenesis	2/26	0.0346

**Table 9:** Differential lipid classification with significant *P* values (*P* < 0.05) when spaghetti meat was compared with normal chicken breast meat when enriched using differential lipids (*P* < 0.05).

Lipid categories	Abbreviation	Cohen's D- value	*P* value	Significant lipids
Plasmenylphosphatidylcholine	Plasmenyl-PC	−0.6823	< 0.0001	17
Triglyceride	TG	0.3502	< 0.0001	65
Acylcarnitine	AC	−0.5375	0.0004	15
Phosphatidic Acid	PA	−0.8399	0.0005	6
Phosphatidylinositol	PI	−0.5299	0.0016	13
Diglyceride	DG	−0.4264	0.0026	19
Plasmenylphosphatidylethanolamine	Plasmenyl-PE	−0.3775	0.0038	23
Sphingomyelin	SM	0.3325	0.0038	30
Phosphatidylserine	PS	−0.3525	0.0211	19
Ceramide [N-acyl-sphingosine]	Cer[NDS]	0.49715	0.0253	9
Ceramide [N‑hydroxy-sphingosine]	Cer[NS]	0.35351	0.0415	16

### Validation assays

**Fig. 7:**
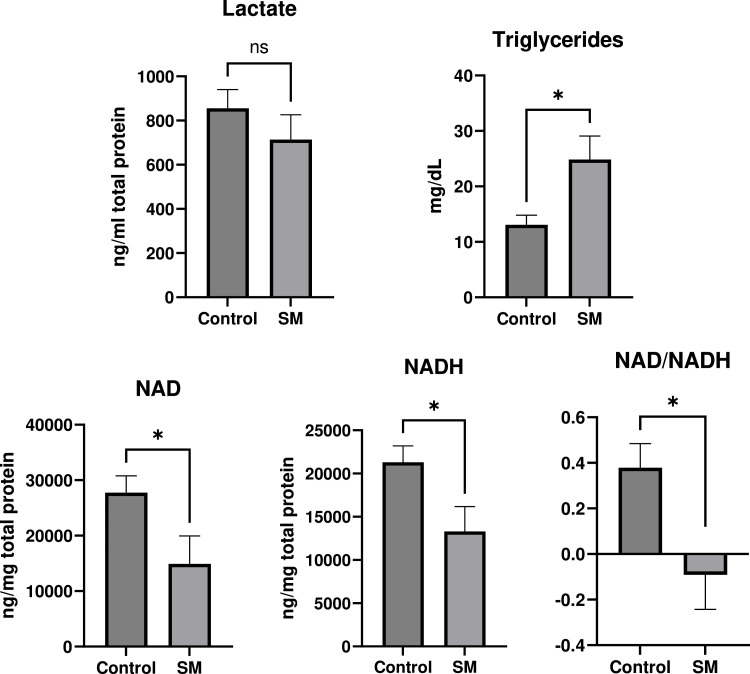
Validation assays for lactate, triglycerides, and nicotinamide adenine dinucleotide (NAD)/ NAD + hydrogen (NADH), in the comparison between spaghetti meat (SM) and normal chicken breast meat (Control).

**Fig. 8:**
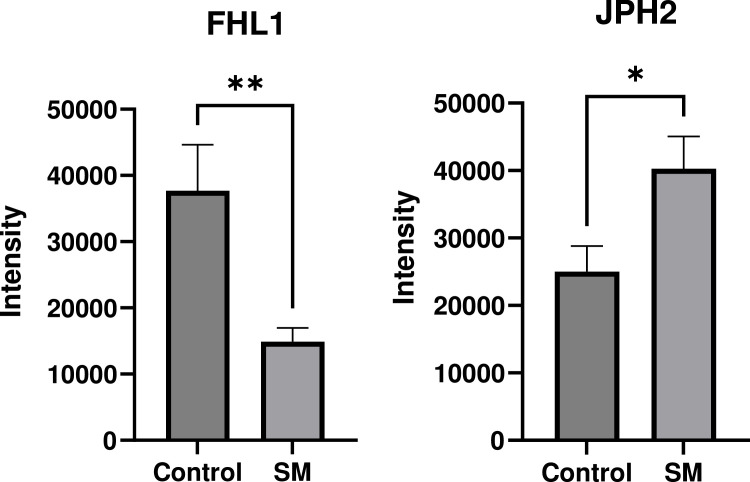
Validation assays for selected proteins (FHL1: four and a half LIM domains and JPH2: junctophilin 2) in the comparison between spaghetti meat (SM) and normal chicken breast meat (Control).

Results for validation assays for lactate, TG, NAD/NADH, and selected proteins (FHL1 and JPH2) were shown in Fig. 7, Fig. 8. There were no significant differences in the lactate level between Control and SM samples (*P* > 0.1). SM had significantly greater levels of TG compared to Control. SM had significantly lower levels of NAD, NADH, and NAD/NADH compared to Control. SM had significantly lower and greater FHL1 and JPH2 protein content, respectively, compared to Control.

## Discussion

The purpose of the study was to investigate differential proteins, metabolites, and lipids in SM compared to Control chicken breast meat. In the current study, a total of 2593 molecules were identified and composed of 1903 proteins, 506 lipids, 181 compounds and 3 electrolytes. Table 10 shows the number of identified proteins, metabolites, and lipids in the untargeted LC/MS proteomics, metabolomics, and lipiomics in previous studies. Using untargeted methods for entire proteins, metabolites, and lipids, these previous studies identified more than 1,000 proteins, 500 metabolites, 400 lipids, respectively. In the current study, a lower number of metabolites were identified compared to the untargeted LC/MS metabolomics methods (Choi et al., 2024a). However, the multiomics approach the current study utilized has the advantage of investigating all molecules in one analysis. By examining all types of molecules simultaneously within the same samples, this approach ensures a more comprehensive understanding of the biological processes and their interactions.

**Table 10:** The number of proteins, metabolites, and lipids identified using untargeted LC/MS proteomics, metabolomics, and lipidomics methods in chicken breast meat in previous studies.

References	Methods	Identified numbers
(Kong et al., 2024)	Untargeted LC/MS proteomics	1051 proteins
(Huang et al., 2022)		1300 proteins
(Zhang et al., 2021)		1413 proteins
(Choi et al., 2024a)	Untargeted LC/MS metabolomics	3090 metabolites
(Shi et al., 2022)		1332 metabolites
(Zhang et al., 2020)		546 metabolites
(Kong et al., 2023)	Untargeted LC/MS Lipidomics	560 lipids
(Lv et al., 2023)		492 lipids
(Wei et al., 2022)		440 lipids

Among the top 25 molecules with the greatest fold changes, the distribution was relatively balanced, consisting of 10 proteins, 7 metabolites, and 8 lipids. However, the overall number of differentially expressed proteins was greater than that of lipids and metabolites. This may suggest that, despite fewer identified lipids and metabolites, the alterations in these molecules were more noticeable in SM compared to Control, whereas proteins showed a broader but less dramatic variation. Still, a previous study by Che et al. (2024) showed there were 4018 differential genes in the comparison of SM and normal chicken breast meat. These results may suggest that there are distinct alterations in genes, proteins, metabolites, and lipids in SM compared to normal breast meat. However, the PCA plot and cluster dendrogram showed that the SM and Control were not clearly separated based on the differential proteins, metabolites, and lipids in the current study. This result is consistent with previous data (Choi et al., 2024a), which demonstrated that SM was not clearly separated from normal chicken breast meat according to the PCA analysis.

Calponin was the most upregulated molecule in SM compared to Control in the current study. Calponin is a calcium binding protein in smooth muscle associated with muscle contraction as it inhibits actomyosin ATPase (Winder and Walsh, 1993). Sasaki et al. (2002) showed that expression of calponin was increased in tumor vessels. Vascular smooth muscle stretch and venous damage may increase the expression of calponin in WB (Mutryn et al., 2015). Velleman et al. (2024) suggested that calponin could be associated with the compromised function of satellite cells in chicken breast muscles. Increased protein expression of calponin in the current study suggests that SM could be related to vascular diseases or SM could have similar etiologies with WB. In the current study, FHL1 decreased and JPH2 increased in SM compared to Control. A previous study by Cowling et al. (2008) showed that FHL1 is related to muscle hypertrophy and upregulated in WB. JPH2 plays an important role in regulating muscle contraction via modulating Ca^2+^ signaling and formatting sarcoplasmic reticulum–plasma membrane junctions in striated muscle (Perni, 2022). Potentially, increased JHP2 in SM could be associated with the detachment of muscle fibers, which is the representative characteristic of SM.

In the current study, NAD decreased in SM compared to Control. Validation assays showed that SM had significantly lower NAD, NADH, and NAD/NADH than Control. NAD is an omnipresent biological molecule that participates in various metabolic reactions such as oxidative phosphorylation and ATP production, DNA repair, gene expression, intracellular calcium signaling, and immunological functions (Lin and Guarente, 2003). According to Braidy et al. (2019), reduced NAD may indicate DNA damage due to oxidative stress and/or inflammation. Previous data (Choi et al., 2024a) consistently showed that NADH (NAD + hydrogen) was decreased in SM. Shakeri et al. (2023) reported a reduced NAD/NADH ratio in WB suggesting a potential link to metabolic disorders. Adenosine 5′-diphosphoribose decreased in SM in the current study. This molecule is one of the metabolites from NAD and has important roles in DNA repair, calcium signaling, diverse metabolic pathways, and energy metabolism (Zhang et al., 2015). Decreased NAD could be associated with reduced adenosine 5′-diphosphoribose in SM. In the current study, ATP synthase membrane subunit f was upregulated in SM. Increased ATP synthase membrane subunit f, which has a crucial role in producing ATP, would be a defensive mechanism to compensate for compromised energy metabolism in SM. Decreased protein‑serine/threonine kinase, which catalyzes the transfer of a phosphate from ATP to a protein substrate (Diallo and Prigent, 2011), is consistent with compromised energy metabolism in SM in the present study. Furthermore, enrichment assays for proteins and metabolites showed that carbon metabolism and energy metabolism were different in SM compared to Control. However, validation assays showed no significant differences between SM and Control in the concentration of lactate, which is a major metabolite generated during ATP production during the postmortem period. This discrepancy could originate from different analytic methods. In WB, compromised carbon metabolism and energy metabolism are considered one of the major etiologies (Kuttappan et al., 2017). Therefore, carbon metabolism and energy metabolism were negatively affected in SM similar to WB.

Decanoylcarnitine, lauroylcarnitine, linoleyl-carnitine, oleoyl-carnitine, hexanoylcarnitine, belonging to AC family, were downregulated, and propionylcarnitine were upregulated in SM compared to Control in the current study. Lipid enrichment assays also showed that AC decreased in SM compared to Control. AC play important roles as carriers to transport activated long-chain fatty acids into mitochondria for β-oxidation as a major energy source for cell proliferation and activities (Li et al., 2019). Reduced AC may indicate that the muscle cells in SM may have disorders in energy supply and usage because reduced AC indicates that β-oxidation, an important process for energy production (Tarasenko et al., 2018), was suppressed. Energy production is important for skeletal development in broiler chickens (Mohammadabadi et al., 2021), and gluconeogenesis and glycolysis are considered as potential etiological factors in the myopathy development, especially WB in chicken breast meat (Kuttappan et al., 2017). Increased propionylcarnitine levels may suggest disorders in mitochondrial function and fatty acid metabolism (Lee and Park, 2023). Moreover, the KEGG enrichment assay in the current study showed that carbon metabolism was decreased in SM. However, in the current study, the TG were increased in SM compared to Control, which was analyzed in multiomcis and colorimetric analyses. An increase in TG, a key form of lipid accumulation (Hermier, 1997), is not preferred in chicken breast meat because consumers prefer lean meats (Liu et al., 2018). TG, composed of a glycerol molecule and three fatty acids, is an important energy source for β-oxidation (Selen et al., 2021). TG were significantly increased and AC were numerically decreased in WB according to a previous study (Liu et al., 2022). In a previous study by Kong et al. (2021), biomarkers related to β-oxidation including AC and 3-hydroxybutyric acid (3-HB) were increased in the plasma of broilers with WB. According to Lake et al. (2022), AC was increased in the plasma of broilers with WB, and Abasht et al. (2016) showed that free carnitines were reduced in muscle with WB. In addition, Wang et al. (2023) showed that ketogenic precursors were increased in the breast muscle of broilers with WB. Overall, in WB, β-oxidation would be increased, which could be one of the etiologies to induce WB. Whereas energy sources such as TG for β-oxidation were increased, the transporters (e.g., carnitines) for β-oxidation decreased in muscle with SM in the current study. This suggests that there may be alterations in β-oxidation and energy metabolism in SM, with a likely increase in β-oxidation. Potentially, β-oxidation could be one of the etiologies for SM and WB. Further studies are needed to investigate whether β-oxidation is increased or decreased in SM by analyzing circulating metabolomics.

In the current study, plasmenyl-PC was decreased in SM compared to Control. Plasmenyl-PC which is a phosphatidylcholine (PC) (e.g., phospholipid and choline molecule) is the most common lipid species in chicken breast meat (Liu et al., 2022). PC is a major structure of the lipid bilayer in plasma cell membranes that covers muscle fibers and has a protective role against the unregulated passage of ions and other charged molecules (Liu et al., 2022). The reduction of PC could be associated with myofiber death and muscle wasting (Valentine et al., 2022) and could be associated with the integrity and detachment of muscle fibers in SM. Moreover, PC is a good source of choline for human consumption (Lewis et al., 2015; Zeisel, 2000). This result may suggest that SM is less nutritious for human consumption compared with normal chicken breast meat.

In the current study, enrichment assays performed using differential proteins and metabolomics, showed that biosynthesis of amino acids, ribosome, cysteine and methionine, histidine metabolism, beta-alanine metabolism, and arginine and proline metabolism were influenced in SM compared to Control. These results suggest that protein and amino acid synthesis and their metabolism were affected in SM compared to control. Consistently, Wu et al. (2024) showed that diverse amino acid metabolism pathways were influenced in SM compared to normal chicken breast meat by conducting untargeted metabolomics. Moreover, Reactome pathway analysis of differential molecules, including proteins, metabolites, and lipids, in SM revealed that nonsense-mediated decay was the most enriched pathway, with carbohydrate metabolism pathways also highly represented. The enrichment of nonsense-mediated decay suggests that genetic alterations may contribute to the development of SM. Consistent with this, our previous metabolomic study showed that SM exhibited compromised carbohydrate metabolism similar to WB (Choi et al., 2024a). Moreover, in the current study, SM had significantly higher protein expression of actin compared to Control. Previous data showed that there were alterations in myofibrillar protein profiles of SM compared to Control (Tasoniero et al., 2022). Therefore, SM would have different muscle composition and structure compared to Control.

In conclusion, SM had altered protein, metabolite, and lipid molecule profiles related to β-oxidation, carbon and energy metabolism, lipid formation, and protein and amino acid metabolism compared to normal chicken breast meat. To reduce the incidence and severity of SM in broiler chickens, targeted interventions should focus on the following: 1) increasing NAD and carnitines levels; 2) lowering triglycerides level; and 3) modulating β-oxidation and carbon and energy metabolism through nutritional, genetic, or systemic approaches.

## Funds

We did not have any external funds.

## Declaration of interests

The authors declare that they have no known competing financial interests or personal relationships that could have appeared to influence the work reported in this paper.
